# Acknowledgement to Reviewers of *High-Throughput* in 2018

**DOI:** 10.3390/ht8010003

**Published:** 2019-01-11

**Authors:** High-Throughput Editorial Office

**Affiliations:** MDPI, St. Alban-Anlage 66, 4052 Basel, Switzerland

Rigorous peer-review is the corner-stone of high-quality academic publishing. The editorial team greatly appreciates the reviewers who contributed their knowledge and expertise to the High-Throughput’s editorial process over the past 12 months. In 2018, a total of 41 papers were published in the journal, with a median time to first decision of 19 days and a median time to publication of 46 days. The editors would like to express their sincere gratitude to the following reviewers for their cooperation and dedication in 2018:
Alekseyev, YuriyMarkiv, AnatoliyAlexander, RebeccaMason-Suares, HeatherAlicea, BradlyMathieu, DidierAmara, NeriMazzotti, FabioBandini, ErikaMeerang, MayuraBauer, Joshua A.Menon, VipinBerteotti, ChiaraMiernyk, Jan A.Brewin, NickMohanta, TapanBroszczak, DanielMorris, DouglasBrown, LaurenceNagai, KouheiCaldarelli, StefanoNaryzhny, StanislavCallebaut, IsabelleNees, MatthiasCannataro, MarioNeogi, UjjwalCasals-Terre, JasminaNovelli, AntonioChun, HongguOsolodkin, DmitryCipak Gasparovic, AnaPal, ChandalDash, BirajaPandey, SantoshDelorme, VincentPapantonis, ArgyrisDeng, PengPekar, StanislavDorado, PedroPerez Tur, JordiEboigbodin, KevinPeter, Emanuel K.Elaiw, Ahmed M.Pham, Thang V.Elsayed, Wael A.Pislariu, CatalinaEl-Seedi, Hesham R.Portelius, ErikEl-Sofany, WalaaPuigvert, Jordi CarrerasFabris, LindaRahmanpour, RahmanFeng, ZhiweiRaikhy, GauravGerber, SusanneRaj, NitinGuarascio, MassimoRajpurohit, SurendraHardiman, GerardRamachandran, VinoyHead, StevenRiar, Amanjot KaurHernandez, WenndyRoland, PochetHertz, Daniel L.Rozycki, BartoszHizel, CandanRuddraraju, Kasi ViswanatharajuHolland, MichaelSachkova, MariaHorvath, DragosSampathi, ShilpaHuang, Hsiao-YunSankaranarayanan, Nehru VijiIadarola, PaoloSantarelli, LoryIkonomopoulou, Maria PSantoni-Rugiu, EricIlagan, Ma. Xenia G.Sengupta, ArjunIno, KosukeShen, LishuangIslam, Mohammad MazharulSmyth, Gordon K.Ito, TakuhiroSpakowicz, DanIvanov, Alexander VStanberry, LarissaJain, VaibhavStephenson, RichardJanga, MadhusudhanaStern, AdiJose, JineySturtevant, JoyJurman, GiuseppeSuarez-Ulloa, VictoriaKadimisetty, KarteekSudhir, Putty-ReddyKajdanowicz, TomaszSumoy, LauroKaliviotis, StathisSun, FengjieKaneko, KimiyukiSun, WeiKaplan, ShaunaSuzurikawa, JunKim, SeokminSztuba-Solinska, JoannaKlein, Matthias S.Tagliaferri, PierosandroKomarova, Natalia V.Thakkar, ShraddhaKostic, TanjaThompson, JeffreyKukreja, MuskanTiziani, StefanoKumar, SunilTullo, ApolloniaKunz, MeikVittorioso, PaolaKuzmin, VictorVogt, AndreasLarson, Nicholas B.Vos, RobinLiby, KarenWang, JunLigterink, WilcoWang, JunjieLinsen, LarsXu, SuowenLopez-Camarillo, CesarZaravinos, ApostolosMahmood, KhalidZhang, RuiyongMao, MengZhang, TianyuMarie-helene, David-CordonnierZhang, Yu


